# Compliance to iron and folic acid supplementation in pregnancy, Northwest Ethiopia

**DOI:** 10.1186/s13104-018-3433-3

**Published:** 2018-05-30

**Authors:** Tesfaye Molla Birhanu, Mequanent Kassa Birarra, Fantahun Ayenew Mekonnen

**Affiliations:** 1 Department of Clinical Pharmacy, School of Pharmacy, College of Medicine and Health Sciences, University of Gondar, Gondar, Ethiopia; 2 Department of Epidemiology and Biostatistics, Institute of Public Health, College of Medicine and Health Sciences, University of Gondar, Gondar, Ethiopia

**Keywords:** Compliance**/**adherence, Iron and folic acid supplementation, Antenatal care, Ethiopia

## Abstract

**Objective:**

Strict compliance to iron and folic acid supplementation is vital for prevention of anemia in pregnancy. However, data are scarce in Ethiopia. So, we conducted this study to assess the level of compliance to iron and folic acid supplementation during pregnancy and its associated factors.

**Results:**

Of 418 women, over half, 231 (55.3%), adhered to the recommended iron and folic acid supplementation. Women who started antenatal care (ANC) follow up early [AOR; 95% CI 2.43 (1.12–5.26)], had more frequent number of ANC visit [AOR; 95% CI 2.73 (1.32–5.61)], took small number of tablets per visit [AOR; 95% CI 3.0 (1.21–7.43)], had history of anemia [AOR; 95% CI 1.9 (1.17–3.12)], and were from urban areas [AOR; 95% CI 2.2 (1.29–3.77)], were more likely to conform to recommended iron and folic acid supplementation. Therefore, there need to be prescription of the lowest possible number of tablets per visit. Furthermore, education targeting on increasing maternal health service utilization need to be in place. There need to also be further research aimed at determining the number of tablets to be prescribed per visit specific to individuals’ background characteristics.

## Introduction

Anemia, a low blood hemoglobin concentration, is an important public health problem affecting all countries with varied impact across population segments. Pregnant women are usually among the most affected groups globally [1, 2]. According to WHO (World Health Organization) report, 38.2% of global and 46.3% of African region pregnant women are affected by anemia [3]. In Ethiopia, the prevalence is 23% [4], which seems lower than the aforementioned global and regional prevalences. However, reports show Ethiopia is among countries in the world where the highest maternal and child mortalities are documented, which may likely be due to poor maternal services utilization like micronutrient supplementation [5].

Maternal anemia is associated with mortality and morbidity of the mother and the baby, including risk of miscarriages, stillbirths, prematurity and low birth weight. It impairs children’s development and learning too, further impacting economic productivity and development [6].

A number of causes are reported to influence the occurrence of anemia during pregnancy. Nevertheless, iron deficiency is the leading cause, constituting 41.8% of the global burden [3]. Consequently, WHO recommended 60 mg iron plus 400 µg folic acid supplementation during pregnancy in areas of iron deficiency anemia (IDA), prevalence of above 40% [7]. However, literature indicate that the proportion of women taking iron and folic acid (IFA) supplementation is much lower, specially, in low and middle income countries, continuing to hinder the prevention of anemia. There are several factors believed to be responsible for not conforming to the recommended iron and folic acid supplementation during pregnancy which would be broadly grouped as: socio-economic, ANC utilization and previous illness etc. [8–12]. In Ethiopia the compliance to iron and folic acid supplementation is between 20.4 and 60% [8–10], which is lower than what it should normally be. Nonetheless, the factors contributing for this low level of compliance have not been well studied in the country. Even no study has been conducted in our study setting so far. Therefore, we conducted this study to find out the level of compliance to iron and folic acid supplementation during pregnancy and its associated factors in Northwest Ethiopia.

## Main text

### Methods

#### Study design and setting

We conducted an institution based cross-sectional study among women attending ANC, and currently taking the supplementation at the University of Gondar Hospital, Ethiopia. The University of Gondar Hospital is one of oldest and largest federally established teaching hospitals. It serves more than 6 million people. The hospital has different specialty units which, included internal medicine, pediatrics, gynecology/obstetrics, surgery, dentistry, psychiatry, ophthalmology, hospital pharmacy and dermatology. The study was conducted from March 8 to April 10, 2017.

#### Sample size and sampling procedure

We determined the sample size using single population proportion formula. We took 60% level of compliance from a previous study [9]. We also considered a 5% level of significance (α) and 5% margin of error. The sample size calculated was, therefore, 384. After adding a non-response rate of 10%, the final sample size obtained was 418 study participants.

The sample women were selected by systematic random sampling from the population attending ANC during the study period. The total number of ANC attendants (sampling frame) for the current study was assumed to be similar to the total number of ANC attendants of the 2 months preceding this study period and it was 2500 attendants. So, we divided 418 (sample) by 2500 (sampling frame) so that a sampling fraction of 1/6 was obtained. To determine the order of the first respondent, we employed simple random sampling technique among the first six participants, and it was found to be the 5th participant. Thus, every 6th participant starting from the first respondent was then included and interviewed until we get the required sample size.

#### Data collection and analysis

The data were collected by interview on variables like, number of iron and folic acid tablets taken per week, reasons for missing tablets, history of anemia status, information provision regarding iron and folic acid supplementation, pregnancy and delivery related characteristics using structured and pretested questionnaire. Compliance to iron and folic acid supplementation was defined as taking iron and folic acid tablets for at least 4 days in the most recent week, which otherwise was considered as non-compliance [7]. Data were checked for consistency and completeness, and then descriptive and analytic computations were carried out. Multivariable binary logistic regression model was fitted to the data to identify predictor variables associated with the dependent variable. Variables with p < 0.05 were considered statistically significant. SPSS version 20 was used to perform the analysis.

#### Ethical consideration

Letter of ethical clearance was obtained from Institution Ethics Review Board of the University of Gondar. Letter of permission was secured from the University of Gondar referral hospital and informed oral consent was obtained from the study participant after providing them with the information concerning the purpose of the study, benefits and harms of participating in the study. The study participants were also told that participation in the study was completely voluntary and the information will be kept strictly confidential.

### Results

#### Socio-demographic characteristics

The mean age of the study participants was 28 ± 5 years. The majority of the respondents were married, 398 (95.2%), and urban residents, 308 (73.7%). Three-fourth of the study participants, 302 (72.2%), were Orthodox Christians. More than half, 227 (54.3%), had four or more household family member. Below half, 192 (45.9%), of the women were house wives and half, 211 (50.5%), of them attended primary or high school education. The average monthly income of study participants was $170 ± 97$ (Table 1).

**Table 1:** Socio demographic characteristics ANC attendants at the University of Gondar hospital, 2017

Variables	Frequency	Percent
Age (years)		
< 28	204	48.8
≥ 28	214	51.2
Marital status		
Married	398	95.2
Single	20	4.8
Residence		
Urban	308	73.7
Rural	110	26.3
Religion		
Orthodox Christian	302	72.3
Muslim	87	20.8
Protestant	29	6.9
Occupation		
Housewife	192	45.9
Government employee	112	26.8
Merchant	64	15.3
Daily laborer	28	6.7
Student	22	5.3
Education status		
Can’t read and write	97	23.2
Primary and high school	211	50.5
College and university	110	26.3
Family size		
< 4	191	45.7
≥ 4	227	54.3
Monthly income		
< $148	245	58.6
≥ $148	173	41.4

#### Compliance to iron and folic acid supplementation

The level of compliance to iron and folic acid supplementation was 55.3%. Around two-third, 142 (61.5%), of the respondents, mentioned advice of the health service providers as a reason for their compliance to the supplement followed by the knowledge they had that iron and folic acid supplementation prevents anemia, 36 (15.5%). The main reason for not taking the supplement as per the recommended, on the other hand, was fear of side effects, 116 (62.0%). Two-fifth, 162 (38.8%), of the respondents collected 30 tablets per ANC visit, and 157 (37.6%) initiated the ANC visit during their first trimester. One-third, 143 (34.2%), had anemia in the current pregnancy (Table 2).

**Table 2:** Compliance to IFA of ANC attendants at the University of Gondar hospital, 2017

Variables	Frequency	Percent
Compliance to supplement		
Yes	231	55.3
No	187	44.7
Reason for compliance		
Advice of health worker	142	61.5
Knowing it prevents anemia	36	15.5
Getting the supplement for free	29	12.6
Use of reminder	24	10.4
Reason for non-compliance		
Fear of side effects	109	58.3
Forget fullness	39	20.9
Too many pills	22	11.8
Unpleasant tests	17	9.0
Tablets collected		
30	162	38.8
60	121	28.9
90	106	25.4
> 90	29	6.9
ANC starting time		
First trimester	157	37.6
Second trimester	219	52.4
Third trimester	42	10.0
Current anemia status		
Yes	143	34.2
No	275	65.8

#### Factors associated with adherence to iron and folic acid supplementation

A total of eleven independent variables were examined for the presence of association with the dependent variable, of which six variables which included, marital status, residence, family number, ANC starting time, facing anemia in the current pregnancy, and number of tablets collected per visit were statistically significantly associated with compliance to iron and folic acid supplementation in the final multivariable logistic regression model (Table 3).

**Table 3:** Predictors of compliance to IFA among ANC attendants at the University of Gondar hospital, 2017

Variables	Compliance		COR, (95% CI)	AOR, (95% CI)
	Yes, no (%)	No, no (%)		
Residence				
Urban	185 (80.1)	123 (65.8)	2.1 (1.35–3.26)	2.2 (1.29–3.77)*
Rural	46 (19.9)	64 (34.2)	1	1
Marital status				
Married	228 (98.7)	170 (90.9)	7.6 (2.19–26.35)	6.3 (1.66–23.89)*
Single	3 (1.3)	17 (9.1)	1	1
Family size				
≥ 4	140 (60.6)	87 (46.5)	1.8 (1.20–2.61)	2.0 (1.16–3.57)*
≤ 3	91 (39.4)	100 (53.5)	1	1
First ANC visit				
First trimester	104 (45)	53 (28.3)	2.4 (1.19–4.74)	2.4 (1.12–5.26)*
Second trimester	108 (46.8)	111 (59.4)	1.2 (0.61–2.29)	1.3 (0.63–2.83)
Third trimester	19 (8.2)	23 (12.3)	1	1
Current anemia status				
Yes	93 (40.3)	50 (26.7)	1.9 (1.22–2.80)	1.9 (1.17–3.12)*
No	138 (59.7)	137 (73.3)		1
Tablet collected				
30	109 (47.2)	53 (28.3)	2.9 (1.3–6.54)	3.0 (1.21–7.43)*
60	59 (25.5)	62 (33.2)	1.2 (0.59–3.06)	1.3 (0.50–3.14)
90	51 (22.1)	55 (29.4)	1.3 (0.57–3.02)	1.0 (0.48–2.64)
> 90	12 (5.2)	17 (9.1)	1	1

“*” Significant at 5% level of significance

### Discussion

This study estimated a 55.3% [95% CI:(50.74%, 60.26%)] level of compliance to iron and folic acid supplementation in ANC attendant women already taking the supplements, and the factors statistically significantly influencing the compliance were marital status, residence, family size, ANC starting time, facing anemia in the current pregnancy, and the number of tablets collected per ANC visit.

The level of compliance identified by this study is consistent with the result in Addis Ababa, Ethiopia (60%) [9], but higher than the findings from other areas of Ethiopia; Mecha (20%) [8], Mishan (39%) [10], and Tigray (33%) [13]. The inconsistency could be due to differences in training level of health care professionals and standard of the health care institution in the different level of health care facilities as this study was conducted on one of the few referral hospitals in the country. This explanation is supported by a study stating high adherence is expected in well-organized setups as adequate counseling and sustainable product availability are better in such facilities [14–17]. However, our finding is significantly lower than from results of studies conducted in India (80.5%) [11] and Bicol Philippines (85%) [12]. This difference may be due to variations in socio-cultural and political environment between the countries [18, 19].

Concerning the factors associated with compliance to iron and folic acid supplementation, the variables which included, marital status, residence, family number, ANC starting time, facing anemia in the current pregnancy, and number of tablets collected per visit were statistically significantly associated. Consequently, women who were urban dwellers were more likely to adhere to their supplements than rural dwellers. This is consistent with a finding of study conducted in Tigray, Ethiopia [13]. This association seems obvious that being urban resident privileges to different enabling factors as compared to rural residents. For instance, urban residents have better access to health facilities [4, 9]. Study participants who had husbands were, similarly, more likely to comply with their supplements compared to their single counter parts. This could be due to the fact that married women gets support from their husband in many ways, like a help in remembering to take the supplement. In addition, higher family number was also associated with better adherence [13]. Women who initiated ANC follow up early was also more likely to adhere to the medicines as compared to those who initiated lately. This result is supported by study done in Bicol Philippines [20]. In the current study, women who had anemia during their recent pregnancy were more adherent than those who did not have anemia. This result is in line with a study from Mecha district, Ethiopia [8]. This may be associated with a better emphasis that might have been payed to those who were sick by health care professionals while providing health education and counseling. It might also be due to a fear in women’s of further complication. In addition, participants who collected few numbers of tablets per visit had higher adherence level. This result is supported by studies from Ethiopia and Egypt [9, 21]. This is because higher number of pills had negative psychological impact on adherence. Studies concluded that as number of tablets decreases adherence to medication increases due to decreasing pill burden [20].

Overall, more than half of the ANC attendant women took the iron and folic acid supplementation as per WHO recommendation. This is an average level of compliance as compared to compliances reported from different literature. Antenatal care visit starting time, number of tablets collected per visit and current anemia status were statistically significantly associated with compliance to the iron and folic acid supplementation in ANC attendants. Therefore, there need to be prescription of the lowest possible number of tablets per visit. Furthermore, education targeting on increasing maternal health service utilization need to be in place. There need also be further research aimed at determining the number of tablets to be prescribed per visit specific to individuals’ background characteristics.

## Limitations

The results of our study might have still be affected by reporting bias since compliance was assessed using self-reported pills intake though the interview required a week-long memory.

## Abbreviations

ANC: antenatal care
EDHS: Ethiopian Demographic and Health Survey
IDA: iron deficiency anemia
WHO: World Health Organization
